# Focal Seizures with Corresponding Neuroimaging and Electroencephalographic Findings in a Patient with Scolex Remnants within a Calcified Cysticercus

**DOI:** 10.4269/ajtmh.18-0400

**Published:** 2018-10

**Authors:** Oscar H. Del Brutto, Naoum P. Issa

**Affiliations:** 1 School of Medicine, Universidad Espíritu Santo—Ecuador, Guayaquil, Ecuador;; 2 Department of Neurology, University of Chicago, Chicago, Illinois

**Figure 1.**
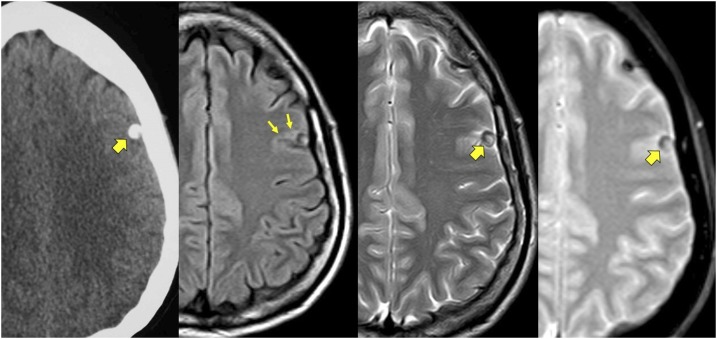
Left panel: Computed tomography showing a superficial single calcified cysticercus in the lower left frontal lobe (thick arrow). Left-central panel: fluid attentuated inversion recovery magnetic resonance imaging sequence showed the calcification surrounded by mild perilesional edema (small arrows). Right-central panel: T2-weighted sequence showing the calcification to be located in a cortical sulcus; the interior of the calcification is heterogeneous because of the presence of scolex remnants (thick arrow). Right panel: Echo-gradient sequence confirming the presence of scolex remnants within the calcification (thick arrow) and showing another solid calcification in the upper ipsilateral frontal lobe. This figure appears in color at www.ajtmh.org.

**Figure 2.**
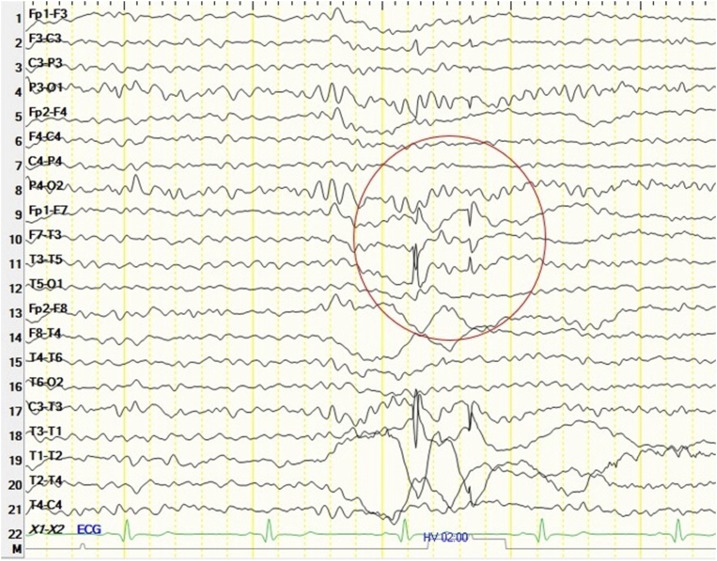
A 21-channel digital electroencephalogram showed focal epileptiform interictal discharges in the left frontotemporal region (circle), corresponding to the location of the calcification with scolex remnants. This figure appears in color at www.ajtmh.org.

A 31-year old woman was admitted to the hospital with a cluster of seizures in the context of a 4-year history of epilepsy. Seizures presented in clusters over 1 or 2 weeks followed by remittances lasting several months. During the clusters, seizures occurred several times a day and always started with involuntary twitching of the right side of the face, sometimes associated with secondary generalization. She had been diagnosed with neurocysticercosis (image not available) at another hospital and started on antiepileptic drugs without improvement. On present admission, neurological examination was unremarkable. Neuroimaging studies showed a calcified cysticercus in the left frontal lobe with heterogeneous content due to presence of scolex remnants (Figure 1). An electroencephalogram (EEG) revealed focal epileptiform interictal discharges corresponding with the location of the calcification (Figure 2). Administration of intravenous corticosteroids rapidly aborted the cluster of seizures.

The association between neurocysticercosis and epilepsy has been demonstrated in large clinical series and population-based studies.^1,2^ However, the lack of correlation between location of parasites, EEG findings, and seizure semiology—noticed in some cases—made some authors to question this relationship, arguing that both conditions may just occur by chance. This report provides proof-of-concept that epilepsy is causally related to neurocysticercosis and reinforces previous studies suggesting that calcified cysticerci may cause recurrent unprovoked seizures when trapped antigenic parasitic remnants get exposed to the host immune system.^3–5^
